# Efficiency and Enhancement in Attention Networks of Elite Shooting and Archery Athletes

**DOI:** 10.3389/fpsyg.2021.638822

**Published:** 2021-03-09

**Authors:** Quanyu Lu, Pengli Li, Qiong Wu, Xinghua Liu, Yanhong Wu

**Affiliations:** ^1^School of Psychological and Cognitive Sciences, Peking University, Beijing, China; ^2^Beijing Key Lab of Learning and Cognition, School of Psychology, Capital Normal University, Beijing, China; ^3^Beijing Key Laboratory of Behavior and Mental Health, Peking University, Beijing, China; ^4^Key Laboratory of Machine Perception (Ministry of Education), Peking University, Beijing, China

**Keywords:** shooting, archery, attention, attention network test, mindfulness training

## Abstract

Attention has been theorized as a system comprising three networks that can be estimated reliably by the attention network test (ANT); the three networks are defined as alerting, orienting, and conflict control. The present study aims to identify the attention networks that are crucial for elite shooting and archery athletes and to examine whether mindfulness training can improve elite athletes' attention networks. We compared the performances in ANT between 62 elite athletes (27 F/35 M, 23.66 ± 4.95 years) from the Chinese national team of shooting and archery and 49 athletes (19 F/30 M, 19.53 ± 3.38 years) from a provincial team in China. The results indicate three well-functioned attention networks in both groups, but elite athletes in the national team responded faster overall than athletes in the provincial team (Diff = 28.84 ms, *p* = 0.006). The 62 elite athletes in the national team then received mindfulness training with varied periods ranging from 5 to 8 weeks, after which the ANT was re-administered. After mindfulness training, the elite athletes improved in orienting (Diff_spatial_ = 10.02 ms, *p* = 0.018) and conflict control networks (Diff_incon_ = 12.01 ms, *p* = 0.019) compared with their pre-training performances. These results suggest that elite shooting and archery athletes in the national team are more efficient in all three attention networks, which means that they are able to reach the alerting state faster, make better use of environmental information, and suppress interference from distractors more efficiently. Moreover, the orienting and conflict control networks of the elite shooting and archery athletes can be improved by mindfulness training. We conclude that mindfulness practice should be considered as a useful addition to daily training for shooting and archery athletes.

## Introduction

Attention is the cognitive ability to focus on specific stimulus or locations (Goldstein, 2011), and there is a consensus among researchers that attention is important for success in sports (Moran, 2004; Abernethy et al., 2007). For example, Memmert (2009) reviewed the literature on visual attention processes in sport expertise. When considering the effect of attention on sport expertise, one of the key points is that the features of the sport can affect the psychological requirements (Moran, 2004). Take the divided attention mentioned in Memmert's (2009) review for example. It refers to the ability that enables people to attend to two or more information sources, and study shows that it is important for basketball players (Memmert, 2006). This may result from the teamwork feature of basketball, which means that success depends on cooperation among players. However, for self-paced and far-aiming sports, such as shooting and archery, the role of divided attention may not be as important as it is for basketball, since the execution of movements is completed at an athlete's own speed and does not rely on teamwork. This leaves open to exploration the issue of what kinds of attention abilities are important for success in self-paced and far-aiming sports. In the current study, we focus on shooting and archery, typical self-paced and far-aiming sports, to identify the attention abilities that are important for elite athletes and to investigate whether these attention abilities can be improved through intervention. Studies have found that subtle disturbances in the attention process can lead to dramatic changes in performance (Landers, 1980; Druckman and Bjork, 1991). One of the most famous cases in shooting is Matthew Emmons, who missed his target in the final of the 50-m rifle three positions in the 2004 Athens Olympics. The Emmons case may reflect the role of different attention abilities. Despite his high alerting state, he might have misused environmental information to locate his target and failed to exert top-down control to inhibit distractions from another's target. These are all important aspects of attention; namely, as elaborated below, orienting and conflict control networks. Emmons' example once again illustrates the need to identify the specific attention abilities that are important for shooting and archery athletes.

Despite the consensus that attention is important for excellent performance in shooting and archery (Hillman et al., 2000; Tremayne and Barry, 2001; Bu et al., 2019), the specific attention abilities that are crucial for elite shooters and archers remain unclear. Existing studies of attention in shooting and archery focus mainly on arousal/activation or vigilance and most of them use electrophysiological methods. Guillot et al. (2003) defined activation in the general sense as a set of processes needed to improve the ability of organisms to process information and to carry out actions. Vigilance is a preparatory attention state for expected cognitive or behavioral activity and needs sustained readiness to detect and respond to environmental changes (Tremayne and Barry, 2001; Guillot et al., 2003). Tremayne and Barry (2001) used skin conductance (SC) and heart rate (HR) as physiological indices to detect activation and vigilance, respectively. They found that SC and HR slowly reduced before the shot and then rebounded instantly after the shot. In addition to arousal/activation and vigilance, it has also been stressed that highly focused steady attention on the target is important for good performance in shooting and archery (Hillman et al., 2000; Bertollo et al., 2012; Kim et al., 2019). This implies that top-down control, which enables athletes to resist interference from distractions, is important for success. It can be inferred from the above that shooting and archery performance is influenced by various attention abilities. It is necessary therefore to systematically examine the relationship between various attention abilities and the sports of shooting and archery based on well-established attention theory in cognitive psychology from an interdisciplinary perspective.

In the current study, we adopted the attention networks theory proposed by Posner and Petersen (1990) for its theorical significance to shooting and archery and its methodological reliability and convenience. Posner and Petersen (1990) proposed that attention can be conceptualized as a system comprising three networks: alerting, orienting, and conflict control (Petersen and Posner, 2012). The alerting network refers to reaching and maintaining a sustained level of vigilance during tasks, which is similar to the definition of vigilance/activation. The orienting network refers to selecting and prioritizing information from sensory input by choosing a modality or location overtly or covertly. Previous studies have not yet addressed the role of this attention ability, but we believe that it is also important for shooting and archery. The reason is that during the aiming process, shooters and archers need to rely on signals from the body (e.g., proprioception) or information from the environment (e.g., wind direction) to adjust gesture or aiming. The conflict control network refers to resolving conflicts by exerting top-down control. This represents the ability to suppress interference from distractors. For shooting and archery, this top-down conflict control would enable athletes to coordinate their body parts in the goal of aiming and to inhibit distractors (such as the targets of other players). By combining cued reaction times (Posner, 1980) and flanker tasks (Eriksen and Eriksen, 1974), Fan et al. (2002) developed a valid attention network test (ANT) to measure these three networks. Participants are required to determine the orientation of a target arrow that is accompanied by two distractor arrows on both flanker sides. The three networks are manifested by the differences in reaction time to alerting cues (alerting), spatial cues (orienting), and flankers (conflict control). Since its release, the ANT has been widely adopted in various studies for its reliable estimation of the three attentional networks and convenient administration (Rueda et al., 2004; Fan et al., 2005; Williams et al., 2016; Pauletti et al., 2017; Mannarelli et al., 2019; Yang and Xiang, 2019). Investigating the differences in attention networks between athletes at different professional levels, such as between national and provincial teams, will shed light on the question of what kind of attention abilities are important for elite athletes in shooting and archery sports.

After identifying the attention abilities that are important for shooting and archery, a further question would be whether they can be improved through effective methods. Besides sports skills training, there is an increasing tendency to apply psychological interventions in sport to help athletes obtain optimal performance (Bernier et al., 2009; Schwanhausser, 2009; Bortoli et al., 2012; Buhlmayer et al., 2017). Mindfulness training is a strong candidate. According to Kabat-Zinn (2003), mindfulness is “the awareness that emerges through paying attention on purpose in the present moment, and nonjudgmentally to the unfolding of experience moment by moment.” Studies have shown that performance in sport (e.g., shooting, swimming, and diving) can be improved by mindfulness training (Solberg et al., 1996; Gardner and Moore, 2004; Schwanhausser, 2009; John et al., 2011), and some researchers have speculated that attention may be the mechanism of these improvements since attention is also enhanced after mindfulness training (Mardon et al., 2016). A recent study by Bu et al. (2019) also found attention enhancement after a sport-specific mindfulness training program in elite shooting athletes. The task Bu et al. (2019) used to measure athletes' attention level was to sequence numbers that were placed in irregular graphics in a random order (Wang and Yao, 2001). This can be seen as an overall indicator of attention, and the question of which specific attention abilities can be improved by mindfulness training remains to be explored.

In order to examine the attentional mechanism behind mindfulness training, it is helpful to find out initially which aspects of attention are important for elite shooting and archery athletes. However, none of the attention measurements in the shooting or archery studies mentioned above involve all three attention networks. Therefore, the purpose of the present study is to identify the specific attention networks that are crucial for elite shooting and archery athletes and to examine whether these networks can be improved by mindfulness training. By testing the differences in ANT performance between two groups of athletes (national vs. provincial), we aim to identify the crucial attention abilities for shooters and archers. Athletes in the national team then received mindfulness training for 5–8 weeks. By testing the differences in ANT performance before and after mindfulness training, we aim to examine if mindfulness training can effectively improve attention networks in elite shooting and archery athletes. As studies have found that mindfulness training can reliably improve the conflict control network in non-athletes (Elliott et al., 2014; Becerra et al., 2017; He and Wang, 2020), we hypothesized that the conflict control network of elite shooting and archery athletes would improve after mindfulness training.

## Materials and Methods

### Participants

One hundred and eleven shooting and archery athletes participated in our study (46 female, 65 male; age: 21.83 ± 4.83 years; Min_age_ = 14, Max_age_ = 40). Sixty-two of them were elite athletes who came from the Chinese national team of shooting and archery (27 female, 35 male; age: 23.66 ± 4.95 years; Min_age_ = 16, Max_age_ = 40), and 49 came from the Hebei provincial team (19 female, 30 male; age: 19.53 ± 3.38 years; Min_age_ = 14, Max_age_ = 29). Athletes in the national team are selected from across the country as representing the best performers in the sport and are candidates for international competitions; therefore, we treat them as elite athletes. Informed consent was obtained from all athletes before the experiment. This study was approved by the Committee for Protecting Human and Animal Subjects, School of Psychological and Cognitive Sciences, Peking University.

### Procedure

All athletes completed the ANT. The 62 elite athletes in the national team then received mindfulness training. The training period varied from 5 to 8 weeks. After the mindfulness training, the ANT was administered again for the elite athletes, giving pre- and post-ANT performance indicators for the elite athletes.

**Figure 1:**
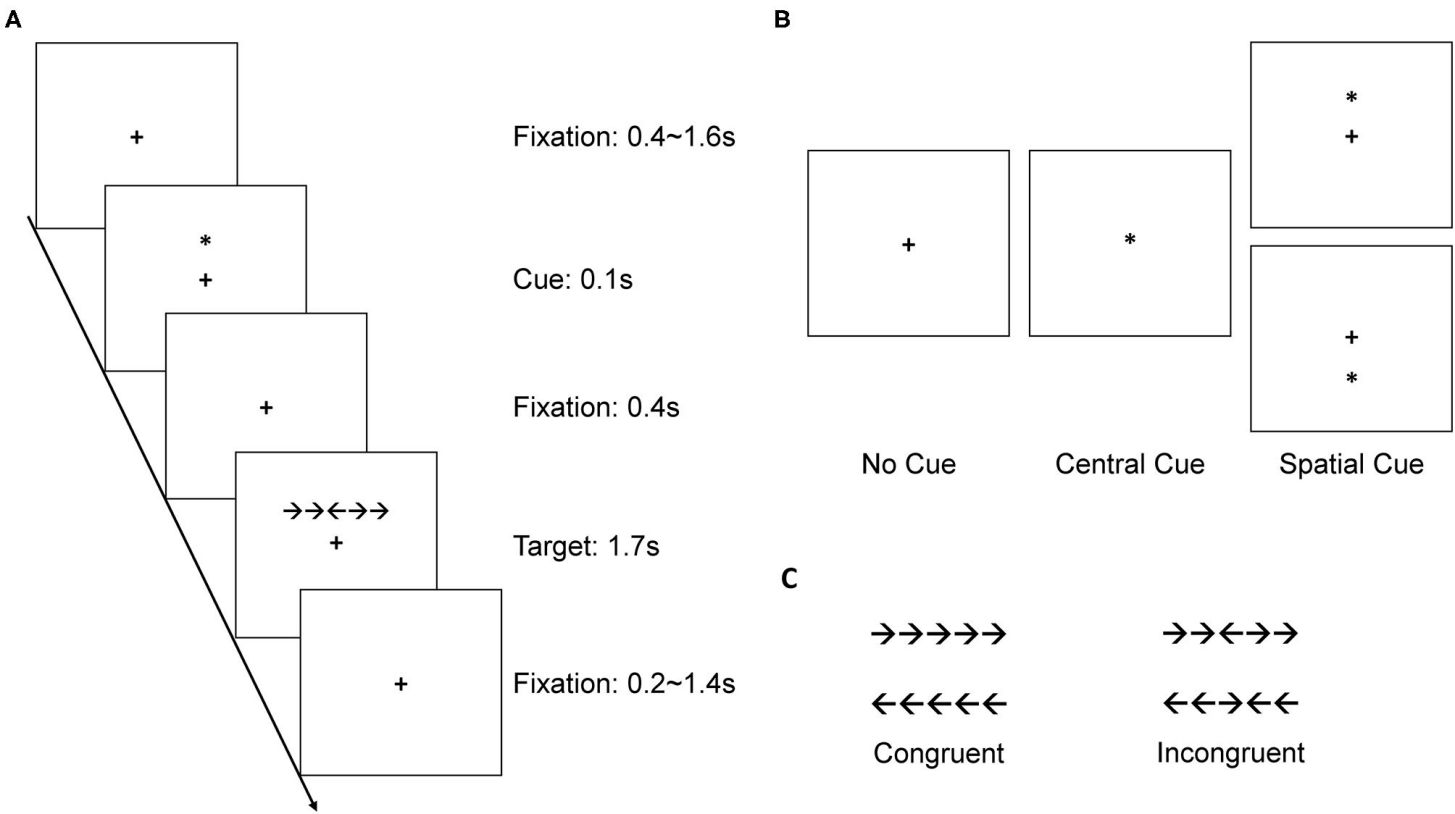
**(A)** Sample trial in the attention network test (ANT). **(B)** Three cue types. **(C)** Four types of target.

The ANT (Figure 1) aims to measure the three functional components of attentional networks: alerting, orienting, and conflict control (Fan et al., 2002, 2005). In each trial, as shown in Figure 1A, a fixation cross would first appear for 0.4–1.6 s, then one of the three types of cue would appear for 0.1 s. Following the cue, after a 0.4 s interval fixation, a target arrow array appeared above or below the fixation for 1.7 s, and participants had to respond within 1.7 s after the target's onset. Finally, a post-target fixation appeared for 0.2–1.4 s to make the total duration of each trial 4 s. The target arrow array consisted of five arrows oriented and arranged horizontally. Participants had to judge the orientation of the center arrow (left or right) and pressed either the left mouse button or the right mouse button accordingly. The two arrows on each flanker side were oriented in either the same direction as the center arrow (congruent condition) or the opposite direction (incongruent condition), as shown in Figure 1C. The three cue types were no cue, central cue, and spatial cue (Figure 1B). In no cue, nothing appeared. In central cue, an asterisk appeared at the fixation cross, indicating that a target arrow array would soon show up but that its location remained unknown. In spatial cue, an asterisk appeared above or below the fixation cross, indicating that a target arrow array would soon show up where the asterisk appeared. The formal experiment began after 12 practice trials and was divided into two parts with an interval. In each part, there were 96 trials, consisting of 32 trials for each cue-type condition. Congruent and incongruent trials were equal under each cue-type condition. The orientations of center arrows to left or right were balanced and all trials were presented randomly across the experiment. The alerting effect was revealed by the differences in reaction time (RT) between no cue and central cue conditions, and orienting effect was revealed by the RT difference between central cue and spatial cue conditions. The conflict control effect was revealed by the RT difference between two flanker types (congruent and incongruent conditions).

The mindfulness training was adapted from mindfulness-based stress reduction (MBSR; Kabat-Zinn, 1990) and mindfulness-based cognitive therapy (MBCT; Teasdale et al., 2000), including sweeping, mindfulness of breath, and other perceptions. Athletes were led by a professional coach and conducted mindfulness practice once a week for 1.5 h. In addition, they took guided homework practice for 15 min every day whenever they were free.

### Data Analysis

SPSS 21.0 was used for data analysis. Overall response accuracy and RTs (ms) were calculated for each group. RTs for each condition of flanker type and cue type were calculated. As there were 37 shooters and 25 archers in the national team, we first compared accuracy in overall and RTs in each condition between elite shooters and archers with *t* tests to determine whether there was any difference in the attention abilities between the two sports (shooting vs. archery). Then, a 2 (Group: National vs. Provincial) × 3 (Cue type: No vs. Central vs. Spatial) ANOVA was conducted to evaluate the difference in alerting and orienting effects between elite athletes and provincial athletes. A 2 (Group: National vs. Provincial) × 2 (Flanker type: Congruent vs. Incongruent) ANOVA was conducted to evaluate the difference in conflict control effect between national and provincial athletes. Similar ANOVA analyses were conducted to examine the effect of mindfulness training on the elite athletes' attention networks. ANOVA analyses to examine effect of mindfulness training were also conducted for elite shooters and archers separately as supplementary analysis.

## Results

Results of *t* tests on accuracy and RTs between elite shooters and archers in the national team did not reveal any significant differences (Table 1). Thus, we think that it is acceptable to combine the data of elite shooters and archers in the following analysis.

**Table 1:** Accuracy and RTs of elite shooting and archery athletes in the national team.

	**Accuracy**	**RT (ms)**					
		**Overall**	**Flanker type**		**Cue type**		
			**Congruent**	**Incongruent**	**No**	**Central**	**Spatial**
Shooting (*n* = 37)	0.97 (0.03)	507.20 (39.33)	463.68 (36.20)	550.73 (44.97)	542.89 (40.15)	520.51 (42.98)	458.22 (39.42)
Archery (*n* = 25)	0.97 (0.03)	510.27 (41.10)	465.75 (39.84)	554.79 (44.34)	548.77 (44.74)	519.00 (44.90)	463.03 (37.76)
*t*	−0.190	−0.295	−0.212	−0.350	−0.540	0.133	−0.480
*p*	0.850	0.769	0.833	0.727	0.591	0.895	0.633

*df = 60*.

### Differences in Attention Networks Between Groups

There were no differences in overall response accuracy between national and provincial teams, *t*_(109)_ = −1.464, *p* = 0.146, Acc_national_ = 0.97 (*SD* = 0.03), Acc_provincial_ = 0.98 (*SD* = 0.02), Diff = −0.01. The overall RT of the national team was shorter than that of the provincial team, *t*_(72.908)_ = −2.621, *p* = 0.011, RT_national_ = 508.44 ms (*SD* = 39.75), RT_provincial_ = 537.28 ms (*SD* = 68.46), Diff = −28.84 ms.

For alerting and orienting effects (Table 2), ANOVA results showed a group effect, *F*_(1,109)_ = 7.723, *p* = 0.006, partial η^2^ = 0.066, *M*_national_ = 508.44 ms (*SD* = 39.75), *M*_provincial_ = 537.28 ms (*SD* = 68.46), and a cue-type effect (Greenhouse–Geisser corrected), *F*_(1.767, 192.556)_ = 999.630, *p* < 0.001, partial η^2^ = 0.902, *M*_no cue_ = 557.90 ms (*SD* = 56.10), *M*_central cue_ = 532.26 ms (*SD* = 57.85), *M*_spatial cue_ = 473.36 ms (*SD* = 57.43). *Post hoc* analysis of cue type indicated significant differences between any two of the three cue-type conditions, all *p* < 0.001. However, the Group × Cue-type interaction did not reach a significant level, *F*_(2,218)_ = 0.123, *p* = 0.865, partial η^2^ = 0.001.

**Table 2:** RT differences between national and provincial teams in each condition.

	**Overall**	**Flanker type**		**Cue type**		
		**Congruent**	**Incongruent**	**No**	**Central**	**Spatial**
National (*n* = 62)	508.44 (39.75)	464.51 (37.40)	552.37 (44.40)	545.26 (41.80)	519.90 (43.41)	460.16 (38.52)
Provincial (*n* = 49)	537.28 (68.46)	491.07 (64.29)	583.50 (74.46)	573.90 (67.26)	547.90 (69.48)	490.05 (71.86)
Difference	−28.84^**^	−26.56^*^	−31.13^*^	−28.64^*^	−28.00^*^	−29.89^*^

* *p < 0.05*,

** *p < 0.01*.

For conflict control effect (Table 2), ANOVA results showed a group effect, *F*_(1,109)_ = 7.723, *p* = 0.006, partial η^2^ = 0.066, *M*_national_ = 508.44 ms (*SD* = 39.75), *M*_provincial_ = 537.28 ms (*SD* = 68.46), and a flanker-type effect, *F*_(1,109)_ = 1,762.865, *p* < 0.001, partial η^2^ = 0.942, *M*_congruent_ = 476.24 ms (*SD* = 52.49), *M*_incongruent_ = 566.11 ms (*SD* = 61.27). However, the Group × Flanker-type interaction did not reach a significant level, *F*_(1,109)_ = 1.134, *p* = 0.289, partial η^2^ = 0.010.

For all three attention networks, the results still hold after controlling for age.

### Effects of Mindfulness Training on Attention Networks

There were no differences in overall response accuracy and RT before and after mindfulness training. For overall response accuracy, *t*_(61)_ = 0.232, *p* = 0.817, Acc_pre_ = 0.97 (*SD* = 0.03), Acc_post_ = 0.97 (*SD* =0.06); and for RT, *t*_(61)_ = 1.595, *p* = 0.116, RT_pre_ = 508.44 ms (*SD* = 39.75), RT_post_ = 501.10 ms (*SD* = 36.55), Diff = 7.34 ms.

For alerting and orienting effects (Table 3), ANOVA results showed a cue-type effect (Greenhouse–Geisser corrected), *F*_(1.815, 110.691)_ = 1046.460, *p* < 0.001, partial η^2^ = 0.945, *M*_no cue_ = 544.05 ms (*SD* = 38.94), *M*_central cue_ = 515.11 ms (*SD* = 42.28), *M*_spatial cue_ = 455.15 ms (*SD* = 38.02), and a Time × Cue-type interaction, *F*_(2,122)_ = 4.669, *p* = 0.013, partial η^2^ = 0.071. *Post-hoc* analysis of cue type indicated significant differences between any two of three cue-type conditions, all *p* < 0.001. Simple effect analysis showed that the RT difference between pre and post mindfulness training reached a significant level only in the spatial condition, Diff_spatial cue_ = 10.02 ms, *p* = 0.018, Diff_no cue_ = 2.42 ms, *p* = 0.638, Diff_central cue_ = 9.574 ms, *p* = 0.076. The time effect did not reach a significant level, *F*_(1,61)_ = 2.544, *p* = 0.116, partial η^2^ = 0.040, *M*_pre_ = 508.44 ms (*SD* = 39.75), *M*_post_ = 501.10 ms (*SD* = 36.55).

**Table 3:** RT differences before and after mindfulness training in the national team (*n* = 62).

	**Overall**	**Flanker type**		**Cue type**		
		**Congruent**	**Incongruent**	**No**	**Central**	**Spatial**
Pre	508.44 (39.75)	464.51 (37.40)	552.37 (44.40)	545.26 (41.80)	519.90 (43.41)	460.16 (38.52)
Post	501.10 (36.55)	461.85 (37.18)	540.36 (38.38)	542.84 (36.16)	510.33 (40.90)	450.14 (37.15)
Difference	7.34	2.66	12.01^*^	2.42	9.57	10.02^*^

* *p < 0.05*.

For conflict control effect (Table 3), ANOVA results showed a flanker-type effect, *F*_(1,61)_ = 1,629.077, *p* < 0.001, partial η^2^ = 0.964, *M*_congruent_ = 463.18 ms (*SD* = 37.16), *M*_incongruent_ = 564.36 ms (*SD* = 41.77), and a Time × Flanker-type interaction, *F*_(1,61)_ = 10.339, *p* = 0.002, partial η^2^ = 0.145. Simple effect analysis showed that the RT difference between pre and post mindfulness training reached a significant level only in the incongruent condition, Diff_incongruent_ = 12.01 ms, *p* = 0.019, Diff_congruent_ = 2.66 ms, *p* = 0.568. The time effect did not reach a significant level, *F*_(1,61)_ = 2.544, *p* = 0.116, partial η^2^ = 0.040, *M*_pre_ = 508.44 ms (*SD* = 39.75), *M*_post_ = 501.10 ms (*SD* = 36.55).

### Effects of Mindfulness Training on Attention Networks in Elite Shooters

There were no differences in overall response accuracy before and after mindfulness training. For overall response accuracy, *t*_(37)_ = 0.383, *p* = 0.704, Acc_pre_ = 0.97 (*SD* = 0.03), Acc_post_ = 0.97 (*SD* = 0.08).

For alerting and orienting effects (Table 4), ANOVA results showed a cue-type effect (Greenhouse–Geisser corrected), *F*_(1.711, 61.590)_ = 615.668, *p* < 0.001, partial η^2^ = 0.945, *M*_no cue_ = 539.79 ms (*SD* = 38.73), *M*_central cue_ = 513.78 ms (*SD* = 42.90), *M*_spatial cue_ = 451.25 ms (*SD* = 39.57). Time × Cue-type interaction did not reach a significant level, *F*_(2,72)_ = 2.655, *p* = 0.084, partial η^2^ = 0.069. *Post-hoc* analysis of cue type indicated significant differences between any two of three cue-type conditions, all *p* < 0.001. Simple effect analysis showed that the RT difference between pre and post mindfulness training reached a significant level only in the spatial condition, Diff_spatial cue_ = 13.95 ms, *p* = 0.010, Diff_no cue_ = 6.21 ms, *p* = 0.312, Diff_central cue_ = 13.46 ms, *p* = 0.056. The time effect reached a marginal significant level, *F*_(1,36)_ = 3.940, *p* = 0.055, partial η^2^ = 0.099, *M*_pre_ = 507.20 ms (*SD* = 39.33), *M*_post_ = 496.00 ms (*SD* = 38.61).

**Table 4:** RT differences before and after mindfulness training of elite shooting athletes in the national team (*n* = 37).

	**Overall**	**Flanker type**		**Cue type**		
		**Congruent**	**Incongruent**	**No**	**Central**	**Spatial**
Pre	507.21 (39.33)	463.68 (36.20)	550.73 (44.97)	542.89 (40.15)	520.51 (42.98)	458.22 (39.42)
Post	496.00 (38.61)	456.75 (38.80)	535.25 (40.71)	536.68 (38.11)	507.05 (42.92)	444.27 (39.57)
Difference	11.21	6.93	15.48^*^	6.21	13.46	13.95^*^

* *p < 0.05*.

For conflict control effect (Table 4), ANOVA results showed a flanker-type effect, *F*_(1,36)_ = 911.161, *p* < 0.001, partial η^2^ = 0.962, *M*_congruent_ = 460.22 ms (*SD* = 37.17), *M*_incongruent_ = 542.99 ms (*SD* = 43.01), and a Time × Flanker-type interaction, *F*_(1,36)_ = 4.772, *p* = 0.036, partial η^2^ = 0.117. Simple effect analysis showed that the RT difference between pre and post mindfulness training reached a significant level only in the incongruent condition, Diff_incongruent_ = 15.48 ms, *p* = 0.015, Diff_congruent_ = 6.93 ms, *p* = 0.245. The time effect reached a marginal significant level, *F*_(1,36)_ = 3.940, *p* = 0.055, partial η^2^ = 0.099, *M*_pre_ = 507.20 ms (*SD* = 39.33), *M*_post_ = 496.00 ms (*SD* = 38.61).

### Effects of Mindfulness Training on Attention Networks in Elite Archers

There were no differences in overall response accuracy and RT before and after mindfulness training. For overall response accuracy, *t*_(24)_ = −0.447, *p* = 0.659, Acc_pre_ = 0.97 (*SD* = 0.03), Acc_post_ = 0.98 (*SD* = 0.03); and for RT, *t*_(24)_ = 0.208, *p* = 0.837, RT_pre_ = 510.27 ms (*SD* = 41.10), RT_post_ = 508.65 ms (*SD* = 32.55).

For alerting and orienting effects (Table 5), ANOVA results showed a cue-type effect, *F*_(2,48)_ = 451.376, *p* < 0.001, partial η^2^ = 0.950, *M*_no cue_ = 550.36 ms (*SD* = 37.99), *M*_central cue_ = 517.09 ms (*SD* = 40.83), *M*_spatial cue_ = 460.93 ms (*SD* = 34.38). *Post-hoc* analysis of cue type indicated significant differences between any two of three cue-type conditions, all *p* < 0.001. The time and time × cue-type interaction effect did not reach significant level, time: *F*_(1,24)_ = 0.043, *p* = 0.837, partial η^2^ = 0.002, *M*_pre_ = 510.27 ms (*SD* = 41.10), *M*_post_ = 508.65 ms (*SD* = 32.55); time × cue-type interaction: *F*_(2,48)_ = 1.958, *p* = 0.152, partial η^2^ = 0.075.

**Table 5:** RT differences before and after mindfulness training of elite archery athletes in the national team (*n* = 25).

	**Overall**	**Flanker type**		**Cue type**		
		**Congruent**	**Incongruent**	**No**	**Central**	**Spatial**
Pre	510.27 (41.10)	465.75 (39.84)	554.79 (44.34)	548.77 (44.74)	519.00 (44.90)	463.03 (37.76)
Post	508.65 (32.55)	469.39 (34.00)	547.91 (34.05)	551.95 (31.62)	515.18 (38.06)	458.83 (32.05)
Difference	1.62	−3.64	6.88	−3.18	3.82	4.20

For conflict control effect (Table 5), ANOVA results showed a flanker-type effect, *F*_(1,24)_ = 698.26, *p* < 0.001, partial η^2^ = 0.967, *M*_congruent_ = 467.57 ms (*SD* = 36.33), *M*_incongruent_ = 551.35 ms (*SD* = 38.88), and a Time × Flanker-type interaction, *F*_(1,24)_ = 5.79, *p* = 0.024, partial η^2^ = 0.194. Simple effect analysis on flanker type showed that the RT difference between pre and post mindfulness training did not reach significant level in both congruent and incongruent conditions, Diff_congruent_ = −3.64 ms, *p* = 0.633, Diff_incongruent_ = 6.88 ms, *p* = 0.431. However, simple effect analysis on time showed that RT differences between congruent and incongruent condition both reached significant level before and after mindfulness training, Diff_pre_ = −89.04 ms, *p* < 0.001, Diff_post_ = −78.52 ms, *p* < 0.001. The time effect did not reach a significant level, *F*_(1,24)_ = 0.043, *p* = 0.837, partial η^2^ = 0.002, *M*_pre_ = 510.27 ms (*SD* = 41.10), *M*_post_ = 508.65 ms (*SD* = 32.55).

## Discussion

The current study has identified the attention networks that are important for elite shooters and archers and examined whether these attention networks can be improved by mindfulness training.

The results showed that elite athletes in the national team responded faster overall than athletes in the provincial team, with an average difference of 28.84 ms. Consistent with previous studies (Fan et al., 2002, 2005), both groups showed similar patterns when responding to different cue types and flanker conditions. They all responded faster in the central cue condition than in the no cue condition, and also responded faster in the spatial cue condition than in the central cue condition, indicating a typical alerting effect and orienting effect, respectively. Also, reactions in the congruent flanker condition were faster than those in the incongruent condition, showing a typical conflict control effect. It is worth noting that although both national and provincial team athletes showed normally functioning attention capabilities, the elite athletes responded faster overall. This means that elite shooters and archers have higher efficiency in all three attention networks. They can reach the alerting state faster, make better use of environmental information, and suppress the interference from distractors more efficiently. For self-paced and far-aiming sports like shooting and archery, such advantages may lead to great improvements in performance.

The results of the mindfulness training replicate the finding that attention abilities can be improved by mindfulness training in shooting and archery athletes. In contrast with previous studies (Bu et al., 2019), we specified the attention networks when examining the enhancement effects of mindfulness training on attention. Although the overall response accuracy and RTs of the elite athletes were not enhanced significantly, they did show improvements under certain conditions. After mindfulness training, elite athletes responded faster in the spatial cue condition and incongruent condition. Such improvements indicate that not only conflict control but also orienting ability were enhanced. In other words, mindfulness training helped to inhibit the influence of distractors and helped the athletes to make better use of environmental information. As the major content of mindfulness training is to allocate attention voluntarily (Teasdale et al., 1995), it is reasonable to assume that orienting and conflict control abilities improved through mindfulness training. During the mindfulness practice, shooting and archery athletes were guided in maintaining attention to a particular point, object, or experience (breathing for example) in a non-judgmental way, or to keep focusing on objects that emerged in the field of attention from moment to moment in a detached way (Kabat-Zinn, 1982). Whenever they found themselves drifting off to something else, they were encouraged to redirect attention to the focus. Gradually, by repeated practice, athletes were able to deploy attention more skillfully and flexibly, which enabled them to be better at utilizing information from sensory input, at identifying targets and distractors, and suppressing interference from distractors. Studies of college students have also shown that mindfulness training improves concentration, particularly the ability to sustain attention, to direct attention from one object to another, and to ignore distractors (Bishop et al., 2004; Sears and Kraus, 2009; Tang and Posner, 2009). Our results show a similar effect of mindfulness training on attention in elite shooting and archery athletes. Individual analysis of elite shooters and archers in the national team replicated the results that conflict control network improved after mindfulness training. The improvement of orienting network was inconsistent in elite shooters and archers. This may result from the relatively small sample size of elite archery athletes. Future studies may further investigate the effect of mindfulness training on orienting network in archery.

Orienting and conflict control abilities are important in shooting and archery sports. As Moran (2011) proposed, abilities to direct and control attention are required for skilled athletes in any sport if they wish to obtain success. It may be particularly crucial for self-paced and far-aiming sports, such as shooting and archery. Researchers agree that maintaining a stable and concentrated attention for a long period is important for shooting athletes (Bu et al., 2019). In order to maintain focus for a long period, shooting and archery athletes must be able to adjust postures based on signals from the body and the environment and have the ability to eliminate interference and redirect attention from distractors back to current targets quickly. It should be noted that, in contrast to psychophysiological studies that focus on the time window before trigger pull, we measured attention abilities in general. This should prompt researchers and coaches to pay attention to the general attention abilities of shooting and archery athletes. The ANT task we used in the present study may serve as a tool for selecting elite shooting and archery athletes and monitoring their attention level. Mindfulness training can be an effective way to improve the attention of shooting and archery athletes.

Despite the findings, there are limitations in our study and future research could explore further the relationship of attention networks, mindfulness training, and shooting and archery in at least three ways. Firstly, the mindfulness training of the present study was part of a psychological service program for the Chinese national team of shooting and archery, which was offered only to elite athletes. We think that mindfulness training for elite shooters and archers may give its own contribution to the field. Results indicated that although elite athletes are more efficient in all three attention networks, some specific attention abilities (orienting and conflict control networks) could be further enhanced. However, we admitted that the study would be more rigorous if athletes in the provincial team also received mindfulness training. It is possible that athletes' skill level may influence the effect of mindfulness training. Studies may further examine the effect of mindfulness training on attention by recruiting athletes with different skill levels.

Secondly, the mindfulness training period in our study varied between 5 and 8 weeks. According to Kaufman et al. (2009), in order for mindfulness training to have an influence on athletes' personal development, a period of practice longer than 4 weeks is recommended. In our study, we managed to ensure that each elite athlete received at least 5 weeks' mindfulness training. The meta-analytical review by Buhlmayer et al. (2017) suggested that a 5-week intervention may be sufficient to have an impact on trait mindfulness. Following this suggestion, we believe that the elite athletes in our study did improve their mindfulness level through the intervention. In addition, we tried as best we could to expand the sample size to increase the power of the test. Such a sample (over 100 athletes) is rare in shooting and archery sports research. For example, in a recent study conducted by Bu et al. (2019), there were only five athletes who participated in a case study. Although it is optimal to adopt a randomization of sample when recruiting participants, this may be hard to satisfy in studies involving special participants like ours. However, we cannot rule out the possibility that the varied periods of mindfulness training may affect its influence on attention and athletes who are exposed to longer training period may exhibit better performance. This suggests our study may in fact report a moderate estimate of the effect of mindfulness training. Future studies could examine the influence of mindfulness training on attention in shooting and archery sports by more rigorous longitudinal studies.

Thirdly, since studies have found a relationship between shooting performance and attention, as indicated by arousal/activation or vigilance (Tremayne and Barry, 2001; Guillot et al., 2003), we speculate that attention networks should correlate with athletes' shooting and archery performance. Moreover, there is evidence that mindfulness training can improve both attention (Tang et al., 2007; Chambers et al., 2008; Tang and Posner, 2009; Hodgins and Adair, 2010) and shooting performance (Solberg et al., 1996; John et al., 2011). Given this evidence and the results of the current study, it is reasonable to infer that attention may mediate the relationship between mindfulness training and improved shooting and archery performance. Future studies could test this mediation effect hypothesis by measuring attention networks, mindfulness training, and shooting and archery performance simultaneously to advance our understanding about the effect of attention and mindfulness training on shooting and archery sports.

In conclusion, this study has analyzed the differences in ANT between shooting and archery athletes in the national team and those in a provincial team to identify the specific attention networks that are important for elite shooting and archery athletes. Additionally, we administered mindfulness training for elite athletes to examine whether it can improve these attention networks. The results for 111 athletes (62 in the national team and 49 in a provincial team) showed that the elite players had an overall faster response to the target. This implies that being able to reach the alerting state faster, making better use of environmental information and suppressing interference from distractors more efficiently are crucial contributors to an elite shooting and archery athlete. Moreover, orienting and conflict control can be improved by mindfulness training. An efficient orienting attention network ensures that athletes can select and prioritize information from sensory input more efficiently. The conflict control network helps athletes to eliminate interference from distractors and maintain focus on targets. These important attention abilities identified in the current study can be used as a point of reference and as indicators when selecting athletes. Future studies are expected to examine the mediation effect of attention on the relationship between mindfulness training and improvements in shooting and archery performance.

## Data Availability Statement

The raw data supporting the conclusions of this article will be made available by the authors, without undue reservation.

## Ethics Statement

The studies involving human participants were reviewed and approved by Committee for Protecting Human and Animal Subjects, School of Psychological and Cognitive Sciences, Peking University. Informed consent to participate in this study was provided by the participants' legal guardian/next of kin.

## Author Contributions

QL and PL contributed to the experimental design, data collection and analysis, and drafting the manuscript. QW contributed to the experimental design, data analysis, and revising the manuscript. XL contributed to data collection and overseeing the study. YW conceived of and oversaw the study, and revised the manuscript. All authors contributed to the article and approved the submitted version.

## Conflict of Interest

The authors declare that the research was conducted in the absence of any commercial or financial relationships that could be construed as a potential conflict of interest.
